# Efficacy and Safety of Stellate Ganglion Block for Treating Angina Pectoris: A Systematic Review and Meta-Analysis

**DOI:** 10.1155/cdr/7134878

**Published:** 2025-02-13

**Authors:** Ying Wei, Jian Xiong, Xiao Li, Fayang Ling, Yi Zhao, Yuxin Sun, Jin Yao, Jinqun Hu, Liyuan Yang, Yu Liu, Wenchuan Qi, Fanrong Liang

**Affiliations:** College of Acupuncture-Moxibustion and Tuina, Chengdu University of Traditional Chinese Medicine, Chengdu, China

**Keywords:** angina pectoris, cervical sympathetic block, ischemic heart disease, meta-analysis, stellate ganglion block

## Abstract

**Purpose:** This systematic review and meta-analysis of randomized controlled trials (RCTs) is aimed at assessing the clinical efficacy and safety of stellate ganglion block (SGB) for angina pectoris (AP).

**Methods:** PubMed, Embase, Cochrane Library, Web of Science, Chinese National Knowledge Infrastructure, China Science and Technology Journal Database, and Wanfang databases were comprehensively searched for RCTs investigating SGB treatment for AP. The retrieval time was from the establishment date of each database to October 10, 2024. The Cochrane risk of bias assessment tool was used to determine the methodological quality. Review Manager 5.4 software was employed for data analysis, and GRADEpro GDT software was utilized to evaluate the quality of evidence.

**Results:** Ultimately, six RCTs were included, encompassing 373 patients with angina. The overall methodological quality of the included studies was moderate, with the evaluation of evidence quality showing 12 low-quality and five extremely low-quality studies. The meta-analysis results demonstrated that compared with the control group, the experimental group had lower frequency and duration of AP, visual analog scale score, heart rate, detection rate of S-T segment elevation ≥ 0.1 mV on electrocardiogram (ECG) after 24 h of treatment, detection rate of abnormal T waves on ECG after 24 h of treatment, and S-T segment displacement on ECG after treatment. Furthermore, the experimental group exhibited lower serum Cardiac Troponin I levels, a decreased incidence of acute myocardial infarction (AMI) and rehospitalization, and improved clinical efficacy. However, none of the included studies reported SGB-related adverse events.

**Conclusion:** SGB is effective in alleviating myocardial injury and reducing the incidence of AMI and rehospitalization in patients with AP. Nevertheless, the limited number and relatively low quality of included studies emphasize the requirement for more high-quality research to verify these conclusions.

## 1. Introduction

Angina pectoris (AP), which is one of the common clinical manifestations of coronary heart disease, is a clinical syndrome characterized by recurrent chest pain or discomfort caused by rapid and temporary ischemia and myocardial hypoxia [1, 2]. AP usually presents as a squeezing pain, stuffiness, or a pulling sensation in the chest. However, some patients may not exhibit chest symptoms but instead experience pain or discomfort in the lower jaw, scapula, shoulder, or fingers, while a few cases are mainly characterized by nonspecific symptoms such as fatigue or nausea. AP can be induced or aggravated by emotional excitement, strenuous exercise, cold exposure, and other stimuli, with rest or sublingual ingestion of nitroglycerin providing relief from this pain [3, 4]. AP severely affects left heart function and is considered a high-risk factor for acute myocardial infarction (AMI), heart failure, and sudden cardiac death. Moreover, AP prevalence in middle-aged people in Sweden was estimated to be 3.5% [5], whereas its prevalence was 4.69% and 7.02% in older men and women, respectively, in India [6].

Previous studies have demonstrated that AP prevalence is positively correlated with age, with nearly half of all cases occurring in the older population (> 65 years) [7, 8]. AP is also a precursor to coronary artery disease and commonly co-occurs with diabetes, hypertension, congestive heart failure, and peripheral vascular disease, which can result in further health problems [8]. Additionally, long-term pain in patients with AP can easily lead to anxiety and depression that worsen their mental health and substantially reduce their quality of life [9]. Some patients even succumb to AMI or heart failure. All these severe consequences exert a heavy economic burden on the families of the affected individuals and medical systems [10]. The current clinical treatment guidelines for AP primarily focus on drug control, with the preferred antianginal drugs consisting of short-acting nitrates, *β*-receptor antagonists, and calcium channel blockers [11]. However, these drugs have a limited analgesic effect and fail to meet the clinical analgesic needs, particularly in unstable angina pectoris (UAP) and variant angina. Therefore, studies exploring new and effective therapies for managing AP are urgently required.

The stellate ganglion (SG), also known as the cervicothoracic ganglion, is part of the cervical sympathetic chain and displays a high degree of variability in its morphology. The SG, which derives its name from its irregular star shape, presents as a fusion of the subcervical ganglion and the first thoracic ganglion anterior to the neck of the first rib in approximately 80% of individuals [12, 13]. In terms of its anatomical location, the SG is located within the vertebral artery triangle and bordered externally by the scalene muscle, internally by the longus colli, trachea, and esophagus, and posteriorly by the C7 transverse process and the prevertebral fascia, with the subclavian artery passing below [14]. Stellate ganglion block (SGB) is a treatment method involving the injection of a local anesthetic into the SG to selectively block the sympathetic nerves innervating the ipsilateral head, neck, chest, and upper limb regions [15]. In 2005, an international paper was published on the effectiveness of SGB in treating chronic refractory angina, leading to a rapid wave of research on this approach [16]. Subsequently, SGB has been gradually introduced into clinical practice. In recent years, evidence-based medicine has also demonstrated the benefits of drug injection therapy for circulatory disorders [17]. SGB is now widely used for managing cardiovascular diseases, immune disorders, endocrine diseases, and various pain syndromes [18, 19]. Prior researchers have shown that the cardiovascular effects mediated by SGB are strongly associated with the inhibition of sympathetic nerve activity, improvement of cardiac blood supply, and attenuation of the cardiac stress response [15, 20, 21]. Furthermore, SGB offers remarkable advantages such as high operability, strong pertinence, low levels of patient pain, and reliable curative efficacy. However, the efficacy and safety of SGB in AP treatment are still not corroborated by evidence-based medicine, and data supporting its standardized use are lacking. Therefore, this systematic review and meta-analysis comprehensively collated and analyzed the published randomized controlled trials (RCTs) on SGB treatment for AP. Our aim was to objectively assess the efficacy and safety of SGB in AP treatment to provide a data-driven basis for the clinical application of SGB in patients with AP.

## 2. Material and Methods

This systematic review and meta-analysis followed the Preferred Reporting Items for Systematic Reviews and Meta-Analysis (PRISMA) statement [22]. This systematic review was prospectively registered on PROSPERO (registration number: CRD42023485135).

### 2.1. Inclusion Criteria

#### 2.1.1. Types of Studies

Clinical RCT studies with no restriction on the publication language.

#### 2.1.2. Types of Patients

In the case of patients with AP, a definite clinical diagnosis of AP was established according to the diagnostic guidelines in the National Institute for Health and Clinical Excellence (NICE) guidelines or the report of the International Society and Federation of Cardiology/World Health Organization on AP [23, 24]. Patient inclusion was not restricted by age, sex, region, race, or AP type, whereas those with congenital heart disease, rheumatic heart disease, malignant tumors, and mental illness were excluded.

#### 2.1.3. Types of Interventions

The experimental group received unilateral or bilateral SGB alone or combined with other therapies. In contrast, the control group did not undergo SGB and was primarily administered conventional medical treatment. Moreover, in studies where the experimental group was treated with SGB combined with other therapies, the control and experimental groups only differed in the use or nonuse of SGB, while the remaining conditions were consistent.

### 2.2. Exclusion Criteria

Studies were excluded based on the following exclusion criteria:

1. Studies involving non-RCTs, cohort studies, animal experiments, cellular experiments, case reports, conference abstracts, research protocols, and reviews.
2. Literature unrelated to SGB or AP and lacking angina-related efficacy indicators.
3. Literature incompatible with the purpose of the current review and meta-analysis.
4. Literature without full-text availability or with prominent data gaps that cannot be filled.
5. Duplicate publication of literature data.

### 2.3. Outcome Indicators

Primary outcomes were as follows: (1) AP symptoms (frequency, duration, and pain intensity of AP), (2) electrocardiogram (ECG) findings (including heart rate (HR), the detection rate of S-T segment elevation ≥ 0.1 mV after 24 h of treatment, the detection rate of abnormal T waves after 24 h of treatment, and S-T segment displacement after treatment), (3) level of serum myocardial enzymes (i.e., Cardiac Troponin I (cTnI)), and (4) clinical efficacy. Secondary outcomes included the following: (1) the levels of vital sign parameters (i.e., mean arterial pressure (MAP) and blood oxygen saturation (SpO_2_)), (2) disease improvement (the transition rate from UAP to stable angina pectoris (SAP)), and (3) postoperative cardiovascular- and cerebrovascular-related adverse events (including the incidence of AMI, interventional therapy, stroke, rehospitalization, and death).

### 2.4. Data Retrieval

We attempted to identify all clinical RCT studies on SGB treatment for AP by comprehensively searching PubMed, Embase, Cochrane Library, Web of Science, Chinese National Knowledge Infrastructure (CNKI), China Science and Technology Journal Database (VIP), and Wanfang databases. The retrieval period for eligible literature was from the date of database establishment to October 10, 2024. Simultaneously, we supplemented the search by examining the Chinese Clinical Trial Registry and ClinicalTrials.gov (https://clinicaltrials.gov/), along with manually searching the reference list of the included studies and related reviews to obtain additional eligible literature. The search terms were a combination of subject-specific words with free-text words, including “angina pectoris,” “coronary artery disease,” “precordial pain,” “stellate ganglion bloc⁣^∗^,” and “cervicothoracic ganglion.” Moreover, the search query was adjusted appropriately based on the characteristics of each database.  Table 1 presents the search strategy employed for data retrieval from the PubMed database.

### 2.5. Literature Screening and Data Extraction

All literature was managed using Zotero software. Two researchers (Y.Z. and J.H.) independently screened the literature according to the established literature inclusion criteria and cross-checked the results. Two researchers (Y.Z. and J.H.) independently extracted information from the included literature into Microsoft Excel 2016 and cross-checked the data, mainly consisting of the first author's name, publication year, region, study type, sample size, age, interventions, outcome indicators, and follow-up time. A third researcher (J.Y.) resolved any differences that arose during these steps.

### 2.6. Bias Risk and Quality Assessment

The bias risk in the included studies was evaluated using Review Manager 5.4 software provided by the Cochrane Collaboration. This evaluation was based on the following seven items: random sequence generation, allocation concealment, the blinding of investigators and participants, the blinding of outcome evaluators, incomplete outcome data, selective reporting, and other biases. Two researchers (Y.L. and L.Y.) independently rated each of these items in each article as “low risk,” “unclear,” or “high risk” and cross-validated them. Any disagreements in the ratings were solved by a third researcher (J.Y.). GRADEpro GDT software was employed to assess the evidence quality of the outcome indicators. The evidence quality was rated as “high,” “moderate,” “low,” or “very low” according to their ratings in terms of five aspects, that is, the limitation of the study design, imprecision, inconsistency, indirectness, and publication bias [25].

### 2.7. Data Analysis

Statistical analysis was performed using Review Manager 5.4 software. Different effect sizes were selected according to the data type. Specifically, relative risk (RR) and 95% confidence interval (CI) were employed for binary variables, whereas mean difference (MD) and 95% CI were calculated for continuous variables. The effect model was selected based on the magnitude of heterogeneity, which was estimated by the *χ*^2^ and *I*^2^ tests. Heterogeneity was considered significant at *p* = 0.1 and *I*^2^ = 50%. In particular, the fixed-effects model (FEM) was chosen for the meta-analysis when heterogeneity was low (*p* > 0.1, *I*^2^ < 50%), while the random-effects model (REM) was utilized in the case of high heterogeneity. Subgroup and sensitivity analyses were applied to explore potential sources of heterogeneity. Additionally, descriptive analysis was performed instead of the meta-analysis when heterogeneity was significant. Finally, *p* < 0.05 was considered statistically significant.

## 3. Results

### 3.1. Literature Screening Process and Outcomes

**Figure 1:**
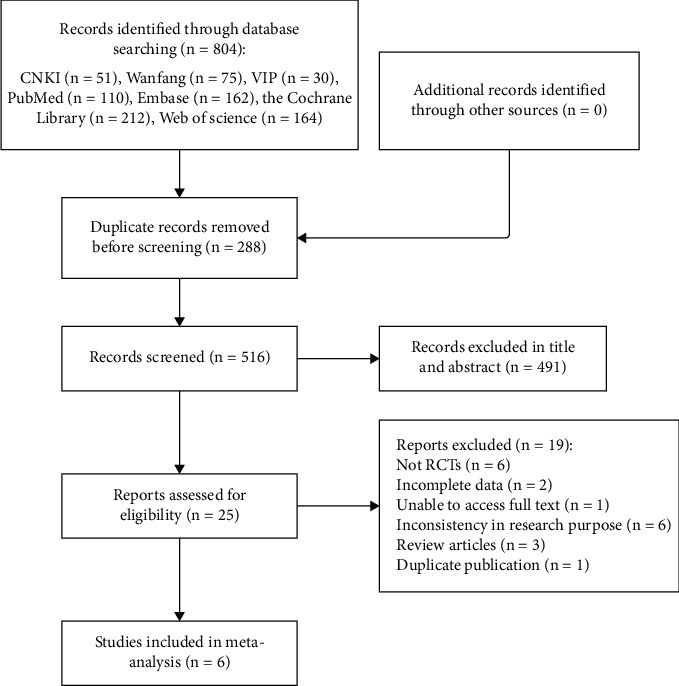
Flowchart illustrating the literature screening process. Abbreviations: CNKI, Chinese National Knowledge Infrastructure; VIP, China Science and Technology Journal Database; RCTs, randomized controlled trials.

According to the established search strategy, we retrieved 804 literature records, with no additional records. After deleting the duplicates, 516 articles were retained. Among them, 491 were excluded after reviewing the titles and abstracts, leaving 25 for further screening. The 491 articles that were excluded after applying our inclusion and exclusion criteria included six case reports, 22 animal experiments, 143 unrelated to AP, 277 unrelated to SGB, two conference abstracts, one scientific and technological achievement, one study protocol, one cell experiment, one news report, 32 reviews, and five meta-analyses. After further reading the full text of the remaining 25 articles, six non-RCT study designs, two articles with incomplete data, one article without full-text availability, six articles with inconsistent research purposes, three reviews, and one duplicate publication were excluded. Ultimately, six articles were included in the meta-analysis [20, 26–30]. The literature screening process is illustrated in Figure 1.

### 3.2. Characteristics of Included Studies

The six included studies were all conducted in China and publicly published in Chinese databases in the years 1996–2011. A total of 373 patients with AP and in the age range of 39–81 years were included. Furthermore, > 60% (230) of the patients had UAP, 65 had drug-resistant AP, and 78 had unspecified AP subtypes. The experimental group comprised 193 patients who were treated with SGB alone, SGB combined with drugs, or SGB combined with conventional medical treatment. In the control group, 180 patients received conventional medical treatment or a single-drug intervention. Additionally, no significant differences were observed between the baseline characteristics across all studies, indicating comparable basic information. The characteristics of the included studies are presented in Table 2.

Finally, one study [28] had clearly misplaced headers and content in two outcome data tables. Hence, we interchanged them appropriately and performed sensitivity analyses to reduce publication bias.

### 3.3. Methodological Quality in Included Studies

**Figure 2:**
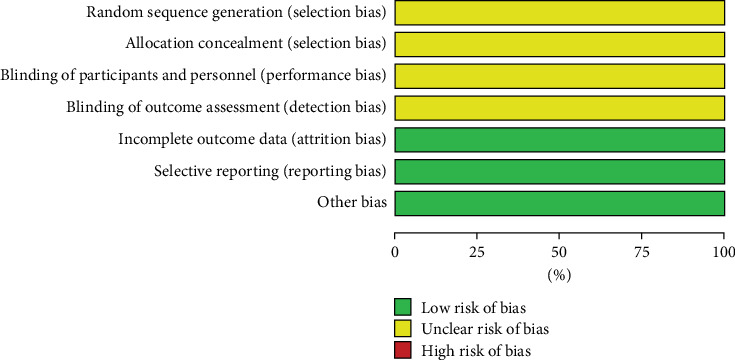
Overall risk of bias assessment of the included studies.

**Figure 3:**
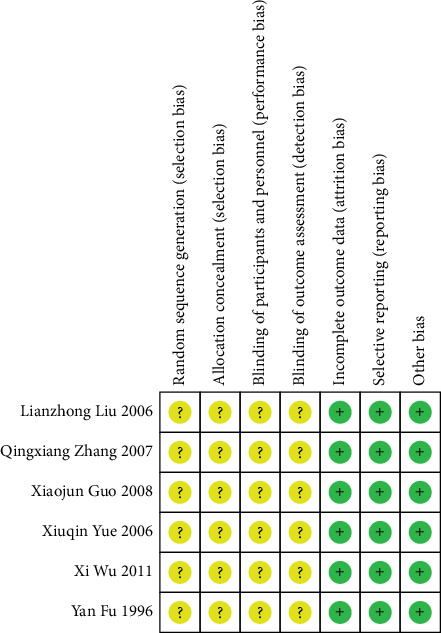
Individual risk of bias assessment chart of the included studies. Note: Green “+” signifies a low risk of bias; yellow “?” denotes an unclear risk of bias.

The Cochrane risk of bias assessment tool was utilized to assess the bias risk in the included studies. The six included studies were all clinical RCTs with no significant differences at baseline. All studies only mentioned randomization but did not specifically describe the method of random sequence generation and allocation concealment nor the implementation of blinding of the investigators, participants, and outcome evaluators. Nevertheless, all studies reported expected outcomes with complete outcome data and a low risk of other biases. The risk of bias assessment is detailed in Figures 2 and 3.

### 3.4. Meta-Analysis Results

#### 3.4.1. Symptoms of AP

##### 3.4.1.1. Frequency of AP

**Figure 4:**
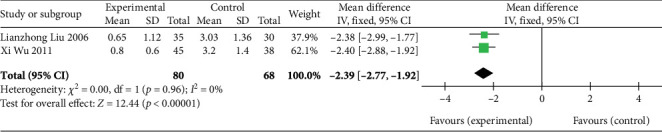
Forest plot of AP frequency in the experimental and control groups. Abbreviations: SD, standard deviation; IV, inverse variance; CI, confidence interval.

Two studies [26, 27] (*n* = 148 participants) reported AP frequency. No significant heterogeneity was found between the two studies (*p* = 0.96, *I*^2^ = 0%); thus, the FEM was selected. The meta-analysis results showed that AP frequency in the experimental group was significantly lower than that in the control group (MD: −2.39, 95% CI: −2.77 to −2.02; *Z* = 12.44, *p* < 0.00001), as depicted in Figure 4. Therefore, SGB can substantially reduce AP frequency.

##### 3.4.1.2. Duration of AP

**Figure 5:**
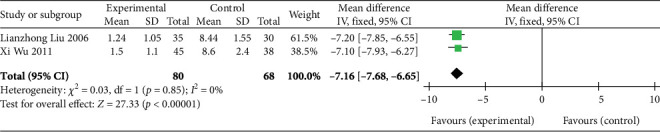
Forest plot of AP duration in the experimental and control groups. Abbreviations: SD, standard deviation; IV, inverse variance; CI, confidence interval.

Two studies [26, 27] (*n* = 148 participants) revealed AP duration. Similar to AP frequency, the FEM was chosen for assessing AP duration due to the nonsignificant heterogeneity (*p* = 0.85, *I*^2^ = 0%). Subsequent analysis demonstrated that the experimental group had a significantly shorter AP duration than the control group (MD: −7.16, 95% CI: −7.68 to −6.65; *Z* = 27.33, *p* < 0.00001), as presented in Figure 5. Thus, SGB has a potential positive effect of shortening AP duration.

##### 3.4.1.3. Pain Intensity of AP

Two studies [20, 29] (*n* = 68 participants) utilized visual analog scale (VAS) scores as a measure of pain intensity in patients with AP. Considering that the VAS scores were reported at multiple time points, we collated the data and conducted a subgroup analysis. Furthermore, the REM was selected owing to the significant heterogeneity between the two studies (*p* < 0.00001, *I*^2^ = 93%). Additional analysis showed significant differences in the VAS scores between the experimental and control groups at 24, 72, 120, and 168 h after treatment (Table 3). These findings imply that SGB can effectively reduce VAS scores and alleviate the pain perception of patients with AP.

#### 3.4.2. ECG Findings

##### 3.4.2.1. HR

Two studies [20, 29] (*n* = 68 participants) provided HR data in patients with AP. Given that multiple time points were involved, data collation and subgroup analysis were performed. Additionally, the FEM was used for analysis because no significant heterogeneity was detected between the two studies (*p* = 0.89, *I*^2^ = 0%). As observed in Table 4, the HR significantly differed between the experimental and control groups at 24, 72, 120, and 168 after treatment. Hence, SGB can adequately reduce the HR of patients with AP.

##### 3.4.2.2. Detection Rate of S-T Segment Elevation ≥ 0.1 mV After 24 h of Treatment

**Figure 6:**
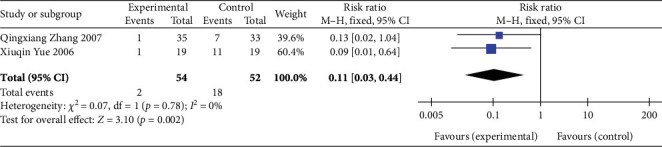
Forest plot of the detection rate of S-T segment elevation ≥ 0.1 mV after 24 h of treatment in the experimental and control groups. Abbreviations: Events, the number of patients with S-T segment elevation ≥ 0.1 mV after 24 h of treatment; M-H test, Mantel–Haenszel test; CI, confidence interval.

Two studies [20, 28] (*n* = 106 participants) reported the detection rate of S-T segment elevation ≥ 0.1 mV after 24 h of treatment. Moreover, considering that the heterogeneity between the studies was relatively small (*p* = 0.78, *I*^2^ = 0%), the FEM was chosen for further analysis. The meta-analysis demonstrated that the detection rate of S-T segment elevation ≥ 0.1 mV after 24 h of treatment was significantly lower in the experimental group than in the control group (RR: 0.11, 95% CI: 0.03–0.44; *Z* = 3.10, *p* = 0.002), as illustrated in Figure 6. These findings suggest that SGB can significantly improve myocardial ischemic injury in patients with AP.

##### 3.4.2.3. Detection Rate of Abnormal T Waves After 24 h of Treatment

Only one study [20] (*n* = 38 participants) evaluated the detection rate of abnormal T waves after 24 h of treatment, demonstrating a significantly lower detection rate in the experimental group than in the control group (RR: 0.15, 95% CI: 0.04–0.59; *Z* = 2.73, *p* = 0.006). Thus, SGB has the potential to effectively reduce AP-induced myocardial damage.

##### 3.4.2.4. S-T Segment Displacement After Treatment

One study [26] (*n* = 83 participants) assessed the S-T segment displacement after treatment, revealing a significant difference between the experimental and control groups (MD: −0.07, 95% CI: −0.10 to −0.04; *Z* = 5.26, *p* < 0.00001). These findings indicate that SGB is beneficial for recovering cardiac function in patients with AP.

#### 3.4.3. Serum Myocardial Enzyme Level

Two studies [20, 29] (*n* = 68 participants) provided data on the level of the serum myocardial enzyme cTnI. In light of multiple time points in the data, we conducted collation and subgroup analysis. Furthermore, FEM analysis showed that the overall combined effect size (MD: −0.27, 95% CI: −0.28 to −0.27; *Z* = 190.49, *p* < 0.00001) was significantly different between the experimental and control groups. Given the significant heterogeneity between the two studies (*p* < 0.00001, *I*^2^ = 100%), REM analysis was performed. This analysis demonstrated an overall combined effect size of MD = −0.30 (95% CI: −0.51 to −0.09; *Z* = 2.78, *p* = 0.005), as presented in Table 5. These results imply that SGB is a potential treatment strategy to reduce the levels of the serum myocardial enzyme cTnI in patients with AP.

#### 3.4.4. Clinical Efficacy

**Figure 7:**
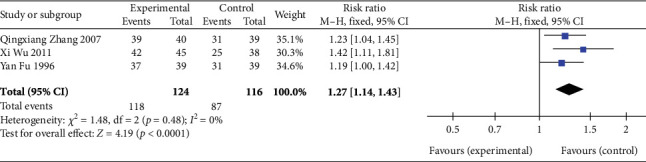
Forest plot of clinical efficacy in the experimental and control groups. Abbreviations: Events, the number of patients who showed treatment efficacy; M-H test, Mantel–Haenszel test; CI, confidence interval.

Three studies [26, 28, 30] (*n* = 240 participants) reported the clinical efficacy. As illustrated in Figure 7, FEM analysis (*p* = 0.48, *I*^2^ = 0%) exhibited significant differences in the clinical efficacy between the experimental and control groups (RR: 1.27, 95% CI: 1.14–1.43; *Z* = 4.19, *p* < 0.0001). Thus, SGB is a promising treatment method for significantly improving clinical efficacy.

#### 3.4.5. Measures of Vital Sign Parameters

##### 3.4.5.1. MAP

Two studies [20, 29] (*n* = 68 participants) measured MAP levels. Considering that the data had multiple time points, we conducted data collation and subgroup analysis. Subsequent FEM analysis (*p* = 0.60, *I*^2^ = 0%) showed that the MAP levels of the experimental group were significantly lower than those of the control group at 24, 72, 120, and 168 h after treatment, as shown in Table 6. Hence, SGB is a valuable approach for reducing MAP levels in patients with AP.

##### 3.4.5.2. SpO_2_

Two studies [20, 29] (*n* = 68 participants) investigated SpO_2_ levels. In view of the multiple time points involved, we conducted data collation and subgroup analysis. The FEM analysis (*p* = 0.19, *I*^2^ = 30%) demonstrated that the SpO_2_ levels in the experimental group were significantly higher than those in the control group at 24, 72, 120, and 168 h after treatment (Table 7). These observations indicate that SGB can effectively enhance SpO_2_ levels in patients with AP.

#### 3.4.6. Disease Improvement

The study by Wu et al. [26] (*n* = 83 participants) examined the transition rate from UAP to SAP to describe the level of disease improvement. The meta-analysis showed an effect size of RR = 1.69 (95% CI: 1.23–2.32; *Z* = 3.22, *p* = 0.001), indicating that SGB could effectively transform UAP into SAP and lead to significant disease improvement.

#### 3.4.7. Postoperative Cardiovascular- and Cerebrovascular-Related Adverse Events

##### 3.4.7.1. Incidence of AMI

**Figure 8:**
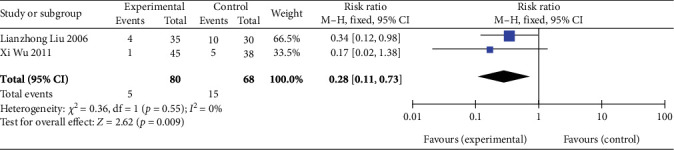
Forest plot of AMI incidence in the experimental and control groups. Abbreviations: Events, the number of patients with AMI; M-H test, Mantel–Haenszel test; CI, confidence interval.

Two studies [26, 27] (*n* = 148 participants) reported AMI incidence during follow-up. Given that no significant heterogeneity was observed between the two studies (*p* = 0.55, *I*^2^ = 0%), FEM analysis was performed. The results revealed an overall combined effect size of RR = 0.28 (95% CI: 0.11–0.73; Z = 2.62, *p* = 0.009), as depicted in Figure 8. This finding indicates that SGB can significantly reduce AMI incidence in patients with AP.

##### 3.4.7.2. Incidence of Interventional Therapy, Stroke, and Rehospitalization

Only one study [27] (*n* = 65 participants) assessed the incidence of interventional therapy, stroke, and rehospitalization during follow-up. The meta-analysis findings suggested that SGB could significantly reduce rehospitalization incidence; however, no such statistical significance was observed for interventional therapy and stroke incidence, as shown in Table 8.

##### 3.4.7.3. Incidence of Death

**Figure 9:**
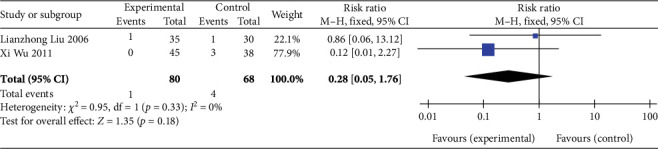
Forest plot of death incidence in the experimental and control groups. Abbreviations: Events, the number of patient deaths; M-H test, Mantel–Haenszel test; CI, confidence interval.

Two studies [26, 27] (*n* = 148 participants) investigated the incidence of death during follow-up. FEM analysis (*p* = 0.33, *I*^2^ = 0%) showed no significant differences in the overall combined effect size between the experimental and control groups (RR: 0.28, 95% CI: 0.05–1.76; *Z* = 1.35, *p* = 0.18), as illustrated in Figure 9. Thus, SGB may not reduce death incidence in patients with AP.

### 3.5. Publication Bias

We did not investigate publication bias because fewer than 10 articles were included in this systematic review.

### 3.6. Safety

SGB-related adverse events were not reported in the six included studies. Preliminary evidence suggests that SGB in AP treatment is associated with relatively fewer adverse reactions and higher safety.

### 3.7. Evaluation of Evidence Quality

The quality of evidence was assessed using the Grading of Recommendations Assessment, Development, and Evaluation (GRADE) system. The assessment revealed that the evidence quality ranged from low to very low, which can be attributed to the small sample sizes used in the included studies and the lack of information on allocation concealment and blinding. Of the various outcome indicators (Table 9), HR, MAP, SpO_2_ level, AP frequency, AP duration, the incidence of AMI and rehospitalization, the transition rate from UAP to SAP, the detection rate of S-T segment elevation ≥ 0.1 mV on ECG after 24 h of treatment, the detection rate of abnormal T waves on ECG after 24 h of treatment, S-T segment displacement on ECG after treatment, and clinical efficacy were low-quality evidence. Additionally, cTnI level, VAS score, and the incidence of interventional therapy, stroke, and death were very low-quality evidence.

## 4. Discussion

AP is a prevalent progressive heart disease, which has been gradually increasing in incidence, particularly in the younger population. The pain in AP not only stems from the ischemic chest pain triggered by reduced coronary perfusion, but it is also closely associated with sympathetic overexcitation [20, 31]. Cardiac sensations are primarily conveyed by visceral sensory nerves. Myocardial ischemia and hypoxia can lead to the heightened excitation of the cardiac sympathetic nerves, which transmit the signals to the amygdala, hypothalamus, and insula to ultimately induce pain in the patients. Additionally, the excitation of the cardiac sympathetic nerves can cause cardiovascular contraction, further aggravating ischemia and hypoxia in the ischemic area of the heart and generating a vicious cycle of ischemia and pain [32, 33]. Ischemic stimulation can also induce the increased production of norepinephrine (NE), nerve growth factors (NGFs), and inflammatory factors, thereby triggering stress and inflammatory responses that further exacerbate myocardial injury and pain perception [18]. Therefore, a safe and efficient approach for blocking the transmission of pain signals and the production of pain-eliciting substances is crucial in AP treatment.

SGB can be performed unilaterally or bilaterally alternating, with a successful procedure reflected by Horner syndrome on the ipsilateral side. This treatment mainly regulates cardiovascular movement, pain transmission, and glandular secretion in the distribution area by inhibiting the function of the preganglionic and postganglionic fibers of the SG. SGB also modulates the functional activities of the autonomic nervous system, endocrine system, and immune system via the hypothalamic mechanism. In recent years, the application of ultrasound imaging technology has augmented the visualization, precision, and safety of SGB and notably reduced adverse events such as neurovascular damage caused by earlier blind exploration in the SGB procedure [34]. Consequently, ultrasound-guided SGB has become one of the most commonly used strategies for clinical pain treatment [35]. Prior studies have highlighted that SGB is primarily employed in AP treatment to inhibit the activity of the sympathetic nervous system and induce the following effects [15, 18, 20, 21, 36–39]: (1) effective alleviation of ischemic chest pain by dilating the coronary artery, increasing cardiac blood perfusion, improving myocardial blood and oxygen supply, and accelerating metabolite excretion; (2) protection of cardiac function by inhibiting overexcited cardiac sympathetic nerves to achieve reduced HR, myocardial contractility, and myocardial oxygen consumption; (3) blocking of the pain signal afference by cardiac sympathetic nerves; (4) regulating the stages of pain processing in the central nervous system and alleviating pain perception by affecting the amygdala, hypothalamus, and insula that have neuronal connections with the SG; and (5) effectively diminishing the cardiac inflammatory response and stress myocardial injury and relieving AP by decreasing the production of pain-inducing substances, such as NGFs, NE, neuropeptides, and inflammatory factors.

In this study, we conducted a systematic review and meta-analysis of six RCT studies (including 373 participants) to evaluate the efficacy and safety of SGB for AP treatment. For this purpose, we examined the differences in the outcome indicators, such as symptoms, ECG findings, serum myocardial enzyme levels, and clinical efficacy, between the experimental and control groups. Our results revealed that SGB significantly reduced VAS scores, AP frequency and duration, HR, the detection rate of S-T segment elevation ≥ 0.1 mV, the presence of abnormal T waves on ECG after 24 h of treatment, and the level of the serum myocardial enzyme cTnI, while it improved clinical efficacy. Moreover, the SpO_2_ levels and the transition rates from UAP to SAP were significantly higher, and the MAP level and the incidence of AMI and rehospitalization were significantly lower in the experimental group than in the control group. Conversely, no significant differences were observed in the incidence of interventional therapy, stroke, and death between the two groups. Therefore, these findings indicate that SGB can effectively treat AP by significantly improving disease symptoms and ECG findings, alleviating myocardial injury, promoting cardiac function recovery, and lowering the occurrence rate of some cardiovascular- and cerebrovascular-related adverse events. However, SGB may not prevent death, stroke, and interventional therapy in patients with AP. The overall methodological quality was moderate based on the Cochrane Handbook items. According to the GRADE evidence rating system, the overall quality of the outcome indicators was low, with 12 (70.59%) indicators identified as low-quality evidence and five (29.41%) as very low-quality evidence. Additionally, given the limited number of included studies and small sample sizes, the obtained results require further validation. Although the absence of SGB-related adverse events in all included studies preliminarily suggests that SGB treatment for AP leads to fewer adverse reactions and increased safety, more high-quality evidence is required in the future to verify this conclusion.

Our systematic review and meta-analysis study has a few limitations that should be considered. (1) The number and sample sizes of the included studies were small, and the outcome indicators were found to be low- or very low-quality evidence, all of which might have rendered the statistical analysis results unreliable. Therefore, future high-quality and large-sample clinical RCTs are essential and may prominently influence the existing evidence and alter the assessment results. (2) The included studies were published as early as 1996, with most conducted from 2006 to 2011. Hence, the included studies may not accurately reflect the current research status of SGB technology. Moreover, five of the included studies [20, 26–29] used blind exploration rather than ultrasound guidance for SGB, while another study [30] even used an earlier electrotherapy technology. These less advanced surgical methods may have led to the underestimation of the efficacy and safety of SGB for AP treatment. (3) The overall methodological quality of the included studies was moderate, with limitations such as suboptimal implementation of allocation concealment and blinding. Additionally, blinding of the investigators and participants may have been challenging because SGB is an invasive treatment, which could have further led to biased results. (4) Certain outcome indicators exhibited significant heterogeneity, possibly due to complex factors such as the methodological quality, sample size, and control interventions of the included studies, along with the type of AP and the age, comorbid condition, and physical health of the participants. Although all the control group interventions comprised conventional medical treatments, their specific regimens may have varied depending on the individual patients. Moreover, the surgical method and point of intervention (unilateral or bilateral) employed in the SGB procedure and the proficiency and accuracy of the operators may have contributed to the heterogeneity in the outcome indicators.

Finally, this study provided some prospects and suggestions for future research. First, multicenter, high-quality, and large-sample RCTs employing the latest surgical methods for SGB (such as ultrasound-guided SGB) for AP treatment are urgently required to verify our results and provide an accurate perspective on the therapeutic effect of SGB on AP at the current technological level. Second, safety should be considered a decisive factor in determining the clinical application value of an intervention. Therefore, future studies should equally focus on observing and accurately recording the safety-related indicators associated with SGB in AP treatment, with maximum possible follow-up to supplement the safety evaluation. Third, the prospective registration of clinical RCT studies is essential for effectively improving research transparency, reducing the risk of publication bias, and avoiding the duplication of effort and resource wastage. Lastly, RCTs should strictly adhere to standardized reporting guidelines, including the Consolidated Standards of Reporting Trials (CONSORT), to ensure research with high-quality methodologies.

## 5. Conclusion

Our systematic review and meta-analysis study suggests that SGB in patients with AP can safely and effectively improve disease symptoms and ECG findings, alleviate myocardial injury, promote cardiac function recovery, and reduce the incidence of AMI and rehospitalization. However, considering the limitations in this meta-analysis, more high-quality RCT studies are essential to validate these conclusions.

## Figures and Tables

**Table 1:** Search strategy and results for PubMed database.

**Search number**	**Query**	**Results**
#1	“Angina Pectoris”[Mesh]	45,090
#2	(((((angina[Title/Abstract]) OR (coronary artery disease[Title/Abstract])) OR (chest Bi[Title/Abstract])) OR (precordial pain[Title/Abstract])) OR (stenocardia⁣^∗^[Title/Abstract])) OR (angor pectoris[Title/Abstract])	159,068
#3	#1 OR #2	173,384
#4	“Stellate Ganglion”[Mesh]	2753
#5	(((((((stellate ganglion bloc⁣^∗^[Title/Abstract]) OR (stellate bloc⁣^∗^[Title/Abstract])) OR (ganglion bloc⁣^∗^[Title/Abstract])) OR (sympathetic[Title/Abstract])) OR (autonomic nerve block[Title/Abstract])) OR (cervicothoracic ganglion[Title/Abstract])) OR (cervicothoracic ganglia[Title/Abstract])) OR (stellate ganglia⁣^∗^[Title/Abstract])	95,952
#6	#4 OR #5	96,969
#7	((randomized controlled trial[Publication Type]) OR (randomized[Title/Abstract])) OR (placebo[Title/Abstract])	1,086,760
#8	#3 AND #6 AND #7	110

**Table 2:** Basic characteristics of the included studies.

**Serial number**	**First author**	**Year**	**Region**	**Age range (years)**	**Sex (male/female)**	**AP subtype**	**Disease course (years)**	**Sample size (experimental group/control group)**	**Intervention**		**SGB method**	**Outcome indicator**	**Follow-up duration (years)**
									**Experimental group**	**Control group**			
1	Lianzhong Liu [27]	2006	Shandong, China	41–69	40/25	Drug-resistant AP	1–2	35/30	Isosorbide mononitrate+SGB (daily on one side, alternating left and right)	Isosorbide mononitrate (20 mg orally, twice daily)	Injection of 8–10 mL of 1% lidocaine by blind exploration	⑨⑩⑫	2
2	Qingxiang Zhang [28]	2007	Guangdong, China	46–78	41/38	UAP	—	40/39	Conventional medical treatment+SGB (daily on one side, alternating left and right)	Conventional medical treatment	Injection of 10 mL of 1% lidocaine by blind exploration	⑥⑪	—
3	Xiaojun Guo [29]	2008	Hebei, China	41–67	17/13	UAP	—	15/15	Conventional medical treatment+right side SGB	Conventional medical treatment	Injection of 10 mL of 1% lidocaine by blind exploration	①②③④⑤	—
4	Xiuqin Yue [20]	2006	Guangzhou, China	39–73	25/13	UAP	—	19/19	SGB (daily on one side, alternating left and right)	Conventional medical treatment	Injection of 10 mL of 1% lidocaine by blind exploration	①②③④⑤⑥⑦	—
5	Xi Wu [26]	2011	Hubei, China	44–81	47/36	UAP	—	45/38	Conventional medical treatment+SGB (daily on one side, alternating left and right)	Conventional medical treatment	Injection of 10 mL of 1% lidocaine by blind exploration	⑧⑨⑩⑪⑫⑬	1
6	Yan Fu [30]	1996	Zhengzhou, China	40–58	42/36	AP	0.5–6	39/39	Conventional medical treatment+right side SGB (once a day for 20 min)	Conventional medical treatment	Immersion in 2% procaine and connection to a direct current induction therapy machine to stimulate the SG	⑪	—

*Note:* ①, heart rate (HR); ②, mean arterial pressure (MAP); ③, blood oxygen saturation (SpO_2_); ④, visual analog scale (VAS) scores; ⑤, Cardiac Troponin I (cTnI) level; ⑥, the detection rate of S-T segment elevation ≥ 0.1 mV on electrocardiogram (ECG) after 24 h of treatment; ⑦, the detection rate of abnormal T waves on ECG after 24 h of treatment; ⑧, S-T segment displacement after treatment; ⑨, AP frequency; ⑩, AP duration; ⑪, clinical efficacy; ⑫, postoperative cardiovascular- and cerebrovascular-related adverse events; ⑬, the transition rate from UAP to stable AP (SAP); —, not mentioned in the article.

Abbreviations: AP, angina pectoris; SG, stellate ganglion; SGB, stellate ganglion block; UAP, unstable angina pectoris.

**Table 3:** Comparison of VAS scores between the experimental and control groups.

**Indicators**	**Experimental group (** **x** ± **s**, **n)**	**Control group (** **x** ± **s**, **n)**	**MD (REM) (95% CI)**	**I** ^2^ ** (%)**	**p**	**Z**	**p**
VAS score (24 h)							
Guo et al. [29]	0.8 ± 0.8, 15	5.5 ± 1.5, 15					
Yue et al. [20]	0.96 ± 1.24, 19	7.32 ± 1.76, 19					
	*n* = 34	*n* = 34	−5.51 (−7.14, −3.89)	84	0.01	6.65	< 0.00001
VAS score (72 h)							
Guo et al. [29]	0.7 ± 0.8, 15	5.2 ± 1.4, 15					
Yue et al. [20]	0.78 ± 1.32, 19	7.38 ± 1.43, 19					
	*n* = 34	*n* = 34	−5.54 (−7.60, −3.49)	92	0.0006	5.28	< 0.00001
VAS score (120 h)							
Guo et al. [29]	0.7 ± 0.9, 15	4.2 ± 1.6, 15					
Yue et al. [20]	0.81 ± 1.26, 19	7.24 ± 1.79, 19					
	*n* = 34	*n* = 34	−4.96 (−7.83, −2.09)	94	< 0.0001	3.39	0.0007
VAS score (168 h)							
Guo et al. [29]	0.8 ± 0.4, 15	3.4 ± 1.4, 15					
Yue et al. [20]	0.91 ± 0.24, 19	7.45 ± 1.37, 19					
	*n* = 34	*n* = 34	−4.58 (−8.44, −0.71)	98	< 0.00001	2.32	0.02
Total	*n* = 136	*n* = 136	−5.15 (−6.28, −4.02)	93	< 0.00001	8.93	< 0.00001

Abbreviations: CI, confidence interval; MD, mean difference; REM, random-effects model; VAS, visual analog scale.

**Table 4:** Comparison of HR between the experimental and control groups.

**Indicators**	**Experimental group (** **x** ± **s**, **n)**	**Control group (** **x** ± **s**, **n)**	**MD (FEM) (95% CI)**	**I** ^2^ ** (%)**	**p**	**Z**	**p**
HR (beats/min, 24 h)							
Guo et al. [29]	72 ± 12, 15	90 ± 12, 15					
Yue et al. [20]	71.68 ± 12.45, 19	90.72 ± 10.48, 19					
	*n* = 34	*n* = 34	−18.60 (−24.17, −13.03)	0	0.86	6.55	< 0.00001
HR (beats/min, 72 h)							
Guo et al. [29]	70 ± 13, 15	92 ± 13, 15					
Yue et al. [20]	70.46 ± 12.78, 19	93.89 ± 13.26, 19					
	*n* = 34	*n* = 34	−22.80 (−28.98, −16.61)	0	0.82	7.22	< 0.00001
HR (beats/min, 120 h)							
Guo et al. [29]	68 ± 12, 15	87 ± 15, 15					
Yue et al. [20]	67.56 ± 11.48, 19	89.76 ± 14.38, 19					
	*n* = 34	*n* = 34	−20.86 (−27.16, −14.56)	0	0.62	6.49	< 0.00001
HR (beats/min, 168 h)							
Guo et al. [29]	73 ± 14, 15	88 ± 15, 15					
Yue et al. [20]	72.52 ± 13.29, 19	88.56 ± 14.62, 19					
	*n* = 34	*n* = 34	−15.60 (−22.35, −8.85)	0	0.88	4.53	< 0.00001
Total	*n* = 136	*n* = 136	−19.56 (−22.63, −16.48)	0	0.89	12.45	< 0.00001

Abbreviations: CI, confidence interval; FEM, fixed-effects model; HR, heart rate; MD, mean difference.

**Table 5:** Comparison of meta-analysis results of cTnI levels using the FEM and REM in the experimental and control groups.

**Indicators**	**Experimental group (** **x** ± **s**, **n)**	**Control group (** **x** ± **s**, **n)**	**FEM analysis results**	**REM analysis results**
cTnI level (*μ*g/L, 24 h)				
Guo et al. [29]	0.046 ± 0.008, 15	0.052 ± 0.004, 15		
Yue et al. [20]	0.039 ± 0.0078, 19	0.49 ± 0.0034, 19		
	*n* = 34	*n* = 34	MD: −0.27, 95% CI: −0.27 to −0.26; *Z* = 178.13, *p* < 0.00001	MD: −0.23, 95% CI: −0.66 to 0.21; *Z* = 1.03, *p* = 0.30
cTnI level (*μ*g/L, 72 h)				
Guo et al. [29]	0.065 ± 0.001, 15	0.07 ± 0.078, 15		
Yue et al. [20]	0.069 ± 0.0026, 19	0.67 ± 0.035, 19		
	*n* = 34	*n* = 34	MD: −0.52, 95% CI: −0.53 to −0.50; *Z* = 69.40, *p* < 0.00001	MD: −0.30, 95% CI: −0.89 to 0.28; *Z* = 1.02, *p* = 0.31
cTnI level (*μ*g/L, 120 h)				
Guo et al. [29]	0.067 ± 0.004, 15	0.076 ± 0.048, 15		
Yue et al. [20]	0.063 ± 0.0064, 19	0.7 ± 0.078, 19		
	*n* = 34	*n* = 34	MD: −0.21, 95% CI: −0.23 to −0.19; *Z* = 20.80, *p* < 0.00001	MD: −0.32, 95% CI: −0.94 to 0.29; *Z* = 1.03, *p* = 0.30
cTnI level (*μ*g/L, 168 h)				
Guo et al. [29]	0.066 ± 0.006, 15	0.078 ± 0.062, 15		
Yue et al. [20]	0.066 ± 0.0058, 19	0.75 ± 0.089, 19		
	*n* = 34	*n* = 34	MD: −0.27, 95% CI: −0.29 to −0.24; *Z* = 21.24, *p* < 0.00001	MD: −0.35, 95% CI: −1.01 to 0.31; *Z* = 1.04, *p* = 0.30
Total (95%)	*n* = 136	*n* = 136	(*p* < 0.00001, *I*^2^ = 100%)	(*p* < 0.00001, *I*^2^ = 100%)
			MD: −0.27, 95% CI: −0.28 to −0.27; *Z* = 190.49, *p* < 0.00001	MD: −0.30, 95% CI: −0.51 to −0.09; *Z* = 2.78, *p* = 0.005

Abbreviations: CI, confidence interval; cTnI, Cardiac Troponin I; FEM, fixed-effects model; MD, mean difference; REM, random-effects model.

**Table 6:** Comparison of MAP levels in the experimental and control groups.

**Indicators**	**Experimental group (** **x** ± **s**, **n)**	**Control group (** **x** ± **s**, **n)**	**MD (FEM) (95% CI)**	**I** ^2^ ** (%)**	**p**	**Z**	**p**
MAP (mmHg, 24 h)							
Guo et al. [29]	78 ± 11, 15	90 ± 10, 15					
Yue et al. [20]	76.32 ± 14.11, 19	91.45 ± 12.33, 19					
	*n* = 34	*n* = 34	−13.39 (−19.00, −7.78)	0	0.59	4.68	< 0.00001
MAP (mmHg, 72 h)							
Guo et al. [29]	80 ± 11, 15	91 ± 13, 15					
Yue et al. [20]	78.78 ± 14.46, 19	91.34 ± 14.29, 19					
	*n* = 34	*n* = 34	−11.73 (−18.00, −5.46)	0	0.81	3.67	0.0002
MAP (mmHg, 120 h)							
Guo et al. [29]	80 ± 10, 15	91 ± 11, 15					
Yue et al. [20]	77.82 ± 13.54, 19	90.32 ± 12.18, 19					
	*n* = 34	*n* = 34	−11.69 (−17.23, −6.15)	0	0.79	4.13	< 0.0001
MAP (mmHg, 168 h)							
Guo et al. [29]	82 ± 9, 15	88 ± 10, 15					
Yue et al. [20]	82.52 ± 11.59, 19	87.46 ± 12.34, 19					
	*n* = 34	*n* = 34	−5.53 (−10.60, −0.45)	0	0.84	2.14	0.03
Total	*n* = 136	*n* = 136	−10.26 (−13.05, −7.47)	0	0.60	7.21	< 0.00001

Abbreviations: CI, confidence interval; FEM, fixed-effects model; MAP, mean arterial pressure; MD, mean difference.

**Table 7:** Comparison of SpO_2_ levels in the experimental and control groups.

**Indicators**	**Experimental group (** **x** ± **s**, **n)**	**Control group (** **x** ± **s**, **n)**	**MD (FEM) (95% CI)**	**I** ^2^ ** (%)**	**p**	**Z**	**p**
SpO_2_ (%, 24 h)							
Guo et al. [29]	92.4 ± 2.3, 15	91.4 ± 1.5, 15					
Yue et al. [20]	92.38 ± 2.43, 19	91.35 ± 1.35, 19					
	*n* = 34	*n* = 34	1.02 (0.09, 1.95)	0	0.97	2.14	0.03
SpO_2_ (%, 72 h)							
Guo et al. [29]	94.9 ± 2.2, 15	92.4 ± 1.8, 15					
Yue et al. [20]	94.48 ± 1.67, 19	92.24 ± 1.33, 19					
	*n* = 34	*n* = 34	2.32 (1.52, 3.12)	0	0.77	5.69	< 0.00001
SpO_2_ (%, 120 h)							
Guo et al. [29]	95 ± 2.6, 15	92.4 ± 1.6, 15					
Yue et al. [20]	94.68 ± 2.38, 19	92.56 ± 1.82, 19					
	*n* = 34	*n* = 34	2.33 (1.31, 3.34)	0	0.65	4.49	< 0.00001
SpO_2_ (%, 168 h)							
Guo et al. [29]	95.5 ± 2, 15	92.4 ± 1.9, 15					
Yue et al. [20]	95.37 ± 1.52, 19	92.35 ± 2.23, 19					
	*n* = 34	*n* = 34	3.05 (2.14, 3.97)	0	0.93	6.54	< 0.00001
Total	*n* = 136	*n* = 136	2.19 (1.74, 2.64)	30	0.19	9.50	< 0.00001

Abbreviations: CI, confidence interval; FEM, fixed-effects model; MD, mean difference; SpO_2_, blood oxygen saturation.

**Table 8:** Comparison of interventional therapy, stroke, and rehospitalization incidence in the experimental and control groups.

**Types of adverse events**	**Experimental group**		**Control group**		**RR, M-H test, FEM, 95% CI**	**Z**	**p**
	**Events**	**Total**	**Events**	**Total**			
Interventional therapy	3	35	7	30	0.37 (0.10, 1.30)	1.56	0.12
Stroke	2	35	3	30	0.57 (0.10, 3.20)	0.64	0.52
Rehospitalization	4	35	12	30	0.29 (0.10, 0.79)	2.40	0.02

Abbreviations: CI, confidence interval; FEM, fixed-effects model; M-H test, Mantel–Haenszel test; RR, relative risk.

**Table 9:** Assessment of evidence quality using the GRADE system.

**Outcome indicators**	**Number of participants (number of studies)**	**Risk of bias**	**Inconsistency**	**Indirectness**	**Imprecision**	**Other considerations**	**Quality of evidence**
AP frequency	148 (2)	Serious^a^	Not serious	Not serious	Serious^c^	None	⊕⊕○○ Low
AP duration	148 (2)	Serious^a^	Not serious	Not serious	Serious^c^	None	⊕⊕○○ Low
VAS score	68 (2)	Serious^a^	Very serious^b^	Not serious	Serious^c^	None	⊕○○○ Very low
HR	68 (2)	Serious^a^	Not serious	Not serious	Serious^c^	None	⊕⊕○○ Low
Detection rate of S-T segment elevation ≥ 0.1 mV on ECG after 24 h of treatment	106 (2)	Serious^a^	Not serious	Not serious	Serious^c^	None	⊕⊕○○ Low
Detection rate of abnormal T waves on ECG after 24 h of treatment	38 (1)	Serious^a^	Not serious	Not serious	Serious^c^	None	⊕⊕○○ Low
S-T segment displacement on ECG after treatment	83 (1)	Serious^a^	Not serious	Not serious	Serious^c^	None	⊕⊕○○ Low
cTnI level	68 (2)	Serious^a^	Very serious^b^	Not serious	Serious^c^	None	⊕○○○ Very low
Clinical efficacy	240 (3)	Serious^a^	Not serious	Not serious	Serious^c^	None	⊕⊕○○ Low
MAP	68 (2)	Serious^a^	Not serious	Not serious	Serious^c^	None	⊕⊕○○ Low
SpO_2_ level	68 (2)	Serious^a^	Not serious	Not serious	Serious^c^	None	⊕⊕○○ Low
Transition rate from UAP to SAP	83 (1)	Serious^a^	Not serious	Not serious	Serious^c^	None	⊕⊕○○ Low
Incidence of AMI	148 (2)	Serious^a^	Not serious	Not serious	Serious^c^	None	⊕⊕○○ Low
Incidence of interventional therapy	65 (1)	Serious^a^	Not serious	Not serious	Very serious^d^	None	⊕○○○ Very low
Incidence of stroke	65 (1)	Serious^a^	Not serious	Not serious	Very serious^d^	None	⊕○○○ Very low
Incidence of rehospitalization	65 (1)	Serious^a^	Not serious	Not serious	Serious^c^	None	⊕⊕○○ Low
Incidence of death	148 (2)	Serious^a^	Not serious	Not serious	Very serious^d^	None	⊕○○○ Very low

*Note:* ⊕⊕○○ means that our confidence in the effect estimate is limited: the true effect may be substantially different from the estimate of the effect. ⊕○○○ means that we have very little confidence in the effect estimate: the true effect is likely to be substantially different from the estimate of effect.

Abbreviations: AMI, acute myocardial infarction; cTnI, Cardiac Troponin I; ECG, electrocardiogram; GRADE, Grading of Recommendations Assessment, Development, and Evaluation; HR, heart rate; MAP, mean arterial pressure; SAP, stable angina pectoris; SpO_2_, blood oxygen saturation; UAP, unstable angina pectoris; VAS, visual analog scale.

^a^No description of allocation concealment and blinding.

^b^ *I* ^2^ ≥ 75%.

^c^Total sample size < 400.

^d^Total sample size < 400 and wide confidence interval (CI).

## Data Availability

This study is based on data extracted from published studies. All the data used are included within this manuscript.
